# The effectiveness of olopatadine hydrochloride eye drops for allergic conjunctivitis: Protocol for a systematic review

**DOI:** 10.1097/MD.0000000000018618

**Published:** 2020-02-14

**Authors:** Yingxin Zi, Yu Deng, Meiqi Ji, Yali Qin, Luqi Nong, Ziqiang Liu, Ming Jin

**Affiliations:** a Beijing University of Chinese Medicine; b Department of Ophthalmology, China-Japan Friendship Hospital, Beijing, China.

**Keywords:** allergic conjunctivitis, olopatadine hydrochloride eye drops, protocol, systematic review

## Abstract

**Background:**

Allergic conjunctivitis (AC) is a multifactorial and common type of ocular surface disease that affects many people. The quality of life for AC patients can be significantly decreased caused by symptoms of ocular itching, swelling, redness, and tearing. Topical antihistaminics, mast cell stabilizers, non-steroidal anti-inflammatory drugs (NSAIDs), and steroids have been widely used to treat AC. Many clinical trials have indicated that olopatadine hydrochloride eye drops can provide quick relief of symptoms and signs. The purpose of this review is to evaluate systematically the effectiveness of olopatadine hydrochloride eye drops for treating AC.

**Methods:**

A systematic review of all of the randomized controlled trials on the effectiveness and safety of olopatadine hydrochloride eye drops for AC will be conducted. We will search PubMed, Web of Science (WOS), EMBASE (OVID), the Cochrane Library, Google Scholar, China National Knowledge Infrastructure (CNKI), China Science and Technology Journal database (VIP), Wanfang Database, and CBM, from the database inception date to October 31, 2019. There are no language or publication status restrictions. Registers of clinical trials, potential gray literature, reference lists of studies, and conference abstracts will also be searched. Two reviewers will independently read the articles, extract the data information, and assess the quality of the studies. Data will be synthesized by a heterogeneity test. The primary outcomes include the main symptom and sign scores before and after treatment, the eye redness index, the presence of eosinophils in the conjunctival scraping. Quality of life, the total treatment efficacy, and safety will be evaluated as the secondary outcomes. RevMan V.5.3 software will be used for the meta-analysis.

**Results:**

The study will provide an objective and normative systematic review to evaluate the effectiveness and safety of olopatadine hydrochloride eye drops for the treatment of AC.

**Conclusion:**

Our review will provide useful information to judge whether olopatadine hydrochloride eye drops is an effective intervention for patients with AC.

**Ethics and dissemination:**

It is not necessary to obtain ethical approval as participants are not involved patients. The protocol and results will be published in a peer-reviewed journal. The systematic review will also be disseminated electronically and in print to help guide health care practice and policy.

**Prospero registration number:**

PROSPERO CRD42019132232.

## 1 Introduction

Allergic conjunctivitis (AC) is an inflammatory disease in which the conjunctiva produces a hypersensitivity immune response to external allergens. It is mainly associated with Immunoglobulin E-mediated type I allergic reactions and T lymphocyte-mediated type IV hypersensitivity. Allergic conjunctivitis is one of the common eye diseases.^[1,2]^ AC can be classified into 5 types: seasonal allergic conjunctivitis (SAC), perennial allergic conjunctivitis (PAC), vernal keratoconjunctivitis (VKC), atopic keratoconiunctivitis (AKC), and giant papillary conjunctivitis (GPC).^[3]^ The incidence of AC is 6% to 30% around the world. In patients with allergic rhinitis (AC), 30% to 71% patients suffered from AC or had conjunctival symptoms.^[4]^ Ocular itching is the most frequent and typical clinical manifestation. Eye redness, eyelid swelling, photophobia, tearing, burning, pain, and foreign body sensation are also the common accompanying symptoms.^[5]^ As a seasonal and recurrent disease, AC, which in severe cases might even lead to visual impairment, not only affects the quality of life of the patients such as daily activities and sleep but also poses adverse impacts on physical and mental status and social functioning.^[6]^ With changes both the indoor and outdoor environment, the prevalence of AC is increasing.^[7,8]^ Management of AC are viewed as a global major public health concern.

Current treatment for AC encompasses both non-pharmacological therapies and pharmacological treatments, including identification and avoidance of allergic triggers and exacerbating factors, vasoconstrictors, mast-cell stabilizers, dual-acting antihistamine/mast-cell stabilizers, non-steroidal anti-inflammatory drugs (NSAIDs), and topical steroids therapies.^[9–11]^

As a dual pharmacological actions drug, olopatadine hydrochloride not only suppress histamine H1 receptors, but also stabilize mast cells.^[12]^ Olopatadine hydrochloride has been widely used in the prevention and treatment of numerous allergic diseases.^[13]^ The population of patients with AC is tending to become younger, and especially includes those with allergic rhinitis (AR).^[14]^ However, no critically designed systematic review to evaluate the effectiveness of olopatadine hydrochloride eye drops for AC has been carried out so far. In this article, we will present the protocol and results of this systematic review. The aim is to assess and appraise all of the clinical evidence on the effectiveness and safety of olopatadine hydrochloride eye drops for AC patients.

## 2 Methods

Our protocol has been registered on PROSPERO (registration number: CRD42019132232).^[15]^ This systematic review protocol will follow the Cochrane Handbook for Systematic Reviews of Interventions and the Preferred Reporting Items for Systematic Reviews and Meta-Analysis Protocol (PRISMA-P) statement guidelines.^[16,17]^

### 2.1 Criteria for study inclusion

#### 2.1.1 Types of studies

Only clinical randomized controlled trials (RCTs) of olopatadine hydrochloride eye drops for AC will be searched.

#### 2.1.2 Types of participants

Patients with AC or AC or SAC or PAC or VKC or AKC or GPC will be included. There will also be no restrictions on age, sex, race, educational or economic status, disease duration, and disease severity.

#### 2.1.3 Types of interventions

Studies that evaluated olopatadine hydrochloride eye drops will be included. Trials that compare olopatadine hydrochloride eye drops with another eye drops will also be included. We will exclude studies in which olopatadine hydrochloride eye drops are not used as a major therapy.

#### 2.1.4 Types of outcome measures

##### 2.1.4.1 Primary outcomes

The primary outcomes will include the following: main symptom scores before and after treatment.^[18]^ The conjunctival hyperemia was measured with a noninvasive ocular surface analyzer.^[9]^ The eosinophils in patients with allergic conjunctivitis was detected by impression cytology.^[19]^

##### 2.1.4.2 Secondary outcomes

The secondary outcome measures are total treatment efficacy rate, quality of life (QOL). The former refers to number of patients whose AC improved. QOL will be evaluated by the Chinese version of Allergic Conjunctivitis Related Quality of Life (CACRQOL) score.

##### 2.1.4.3 Safety outcomes

The incidence and severity of side effects will be used to measure safety outcomes. We will use an adverse event report form to record any unexpected events that occurred during the studies.

### 2.2 Search methods for the identification of studies

PubMed, Web of Science (WOS), EMBASE (OVID), the Cochrane Library, Google Scholar, China National Knowledge Infrastructure (CNKI), China Science and Technology Journal database (VIP), Wanfang Database, and CBM, from the database inception date to October 2019, will be searched. The search strategy is as follows: olopatadine hydrochloride eye drops and allergic conjunctivitis (e.g., seasonal allergic conjunctivitis, perennial allergic conjunctivitis, vernal keratoconjunctivitis, atopic keratoconiunctivitis, and giant papillary conjunctivitis). These search terms will be translated into Chinese to search in the Chinese databases. Table 1 showed the specific search strategy used for PubMed. This will also be adjusted in different databases. Meanwhile, PROSPERO, the International Clinical Trials Registry Platform (ICTRP), and ClinicalTrials.gov will be used to identify systematic reviews or ongoing/completed clinical trials related to olopatadine hydrochloride eye drops and AC. We will also review relevant journals, conference processes, papers, and bibliographies.

**Table 1:**
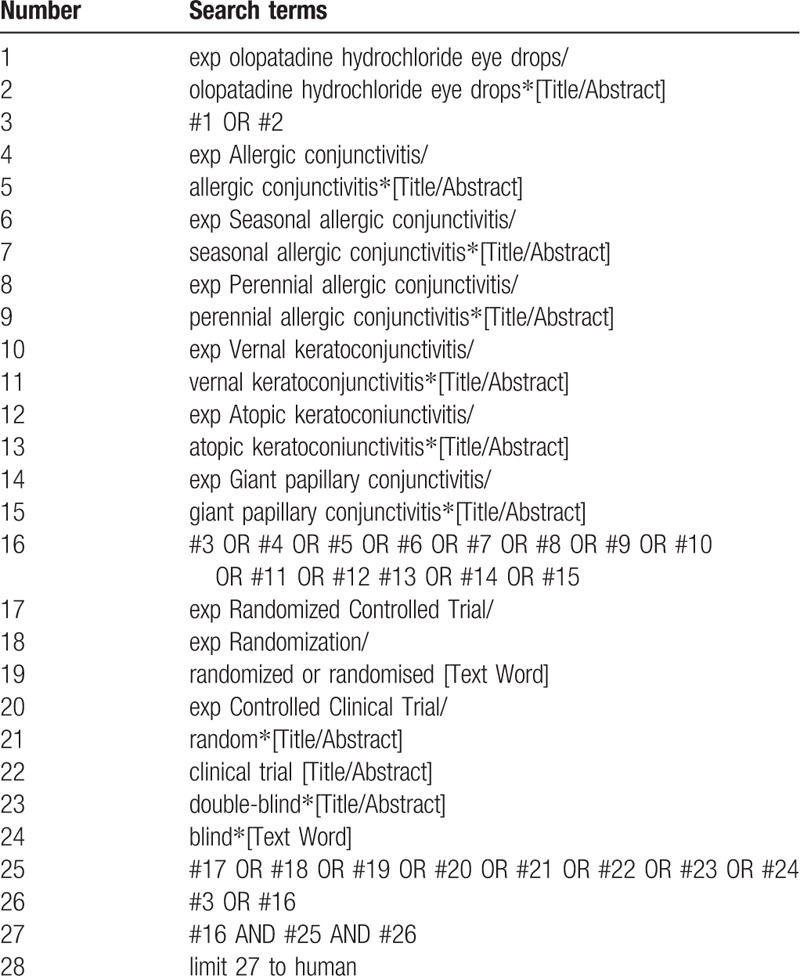
Search strategy used in PubMed database.

### 2.3 Data collection and analysis

#### 2.3.1 Study selection

**Figure 1:**
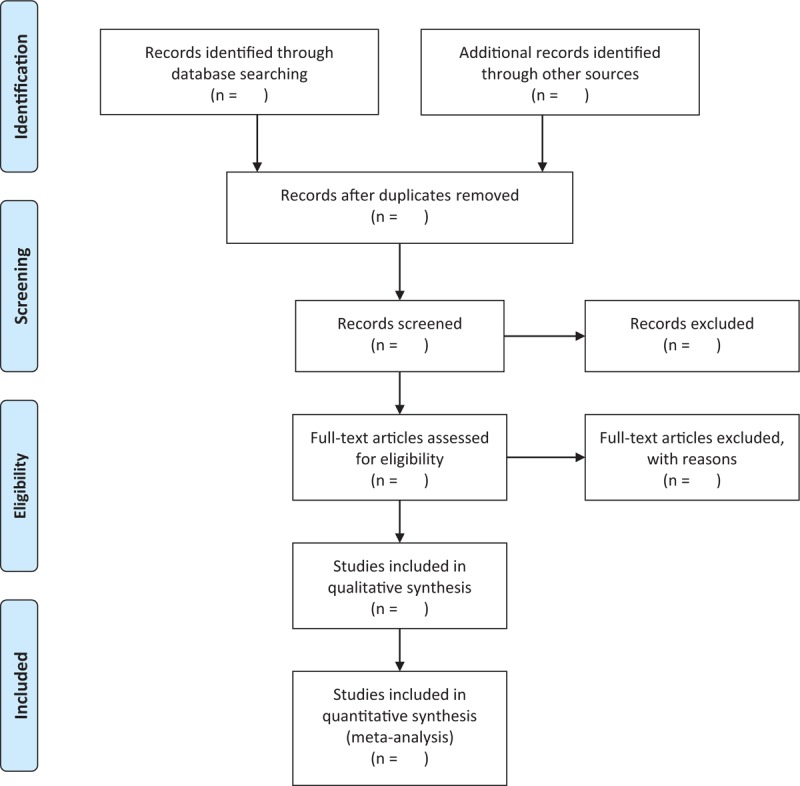
The PRISMA flow chart. PRISMA = Preferred Reporting Items for Systematic Reviews and Meta-Analyses.

Two reviewers (YXZ and YD) will independently search the titles, abstracts, and keywords of retrieved records to include relevant articles. If there are any disagreements between the 2 authors, a third review author (MJ) will conclude discussion between them and give the final decision. The study selection procedure is shown in the PRISMA flow diagram (Fig. 1).

#### 2.3.2 Data extraction and management

Firstly, we will make a standard data collection form according to the inclusion criteria. Secondly, EndNote X9 software (Clarivate Analytics, New York, USA), will be adopted to manage the retrieved records. Thirdly, 2 authors (YXZ and YD) will independently extract data and write on the data collection form. If the authors have any disagreements, a third review (MJ) will take responsibility and make the final decision. If there are lack of the details of the study, the appropriate author will be contacted via e-mail, telephone, or other possible communication means for more useful information. The content of data collection form are as follows:

1. General information: author, title, article types, journal, publication year, volume, period, country, and contact information.
2. Study methods: study design, randomization method, allocation concealment, blinding, sample number, dropout number, incomplete report or selecting report, and other sources of bias.
3. Participants: diagnostic criteria of AC, inclusion criteria, exclusion criteria, age, sex, disease severity, research site.
4. Interventions: olopatadine hydrochloride eye drops, treatment details, treatment duration, and frequency.
5. Outcomes: primary, secondary, and safety outcomes as described above.
6. Notes: ethical approval, financial support, conflicts of interest, and important citations.

#### 2.3.3 Assessment of reporting quality and risk of bias

Two reviews (LQN and ZQL) will independently assess the reporting quality and risk using the risk of bias (ROB) assessment tool in the Cochrane Handbook for Systematic Reviews for Intervention.^[20]^ The Review Manager (RevMan) software (Version 5.0. The Nordic Cochrane Centre, The Cochrane Collaboration, Copenhagen, Denmark) will be used to record relevant details of each trial.^[21]^ The results will be described as “high risk,” “low risk,” and “unclear risk.” We will contact the corresponding author to obtain any basic information that missing for the ROS assessment. A third reviewer (MJ) will make a decisive opinion once there are any discrepancies between 2 authors.

#### 2.3.4 Measures of treatment effect

A risk ratio (RR) and the 95% confidence interval (CI) will be used to analyze dichotomous data and measure the treatment effect. A weighted mean difference (WMD) and a standard mean difference (SMD) with 95% CIs will be used to analyze continuous outcomes. The WMD and SMD will be respectively used for the same assessment instrument and different assessment tools.

#### 2.3.5 Managing missing data

Any missing data may have a potential effect on the final findings of the review, so the review author (HM) will contact the corresponding author or relevant author of an article to obtain more detailed data. If there is no reply from the authors, we will exclude these data and give a detailed explanation in the “Discussion” part.

#### 2.3.6 Assessment of heterogeneity

A standard chi-squared test will be used to assess the heterogeneity.^[22]^ The differences was statistical significance (*P* < .10).^[23]^ The *I*^2^ value will be calculated to quantify the impact of the statistical heterogeneity on the systematic review.

#### 2.3.7 Assessment of reporting biases

When >10 trials are selected, we will use a funnel plot to assess reporting biases.^[24]^

#### 2.3.8 Data synthesis

RevMan (V.5.0) will be used to perform a meta-analysis and to calculate the RR or SMD. We will synthesize and analyze the data according to the level of statistical heterogeneity. If there is no or little statistical heterogeneity, a fixed-effects model will be adopted. When the *I*^2^ value is over 50%, a random effects model will be used for the pooled data. However, if the *I*^2^ value showed considerable heterogeneity in the trials, a meta-analysis will not be performed. Under such circumstance, the source of heterogeneity will be identified from clinical and methodological aspects. We will also present a narrative and qualitative summary. The Grades of Recommendation, Assessment, Development, and Evaluation (GRADE) approach will be used to summarize the findings of the meta-analysis and to describe the strength of the evidence.

#### 2.3.9 Subgroup analysis and investigation of heterogeneity

If there are enough data in the included studies, we will conduct a subgroup analysis to identify heterogeneity. We will obey the following criteria for a subgroup analysis:

1. Type of control interventions, such as vasoconstrictors, mast-cell stabilizers, NSAIDs, topical steroids therapies, other dual-acting antihistamine/mast-cell stabilizers, or no treatment.
2. Treatment number, frequency, and duration.

#### 2.3.10 Sensitivity analysis

After the low quality trials are got rid of, we will use the robustness of a result to conduct a sensitivity analysis. ROB will be used to assess methodological quality. In this situation, a second meta-analysis will be conducted when trials are rated as high risk of bias. Then we will compare and discuss the results and effect size of the 2 meta-analyses.

## 3 Discussion

AC is one of the most common ophthalmological diseases, and its prevalence is ever increasing every year. Currently, antiallergic eye drops include topical antihistamines and mast cell stabilizers can reduce symptoms and signs of AC, which are the mainstay of mild AC therapy.^[25]^ Topical steroids are the main choices for ocular surface inflammations with corneal involvement in severe forms.^[26]^ However, the transient efficacy of these treatments and their potential adverse effects have limited clinical applications.^[9]^ Topical corticosteroids can produce a number of local adverse side effects, for example, cataracts, elevated intraocular pressure (IOP), and increased risk of infection.^[27]^ Thus, olopatadine hydrochloride eye drops for AC are needed to improve efficacy and reduce side effects.^[28]^ As antihistamine and mast cell stabilizer, olopatadine hydrochloride eye drops have been widely used in AC.^[29]^

However, there is lack of long-term data on olopatadine hydrochloride eye drops for AC using evidence-based medicine. Therefore, the purpose of this proposed systematic review and meta-analysis is to evaluate the effectiveness and safety of olopatadine hydrochloride eye drops treatment for AC. We hope that this systematic review will provide more evidence to help patients and clinicians make correct choices when dealing with AC.

## Author contributions

**Conceptualization:** Ying-Xin Zi, Yu Deng, Ming Jin.

**Data curation:** Ying-Xin Zi, Yu Deng, Mei-Qi Ji, Ming Jin.

**Formal analysis:** Ying-Xin Zi, Yu Deng.

**Funding acquisition:** Ying-Xin Zi.

**Investigation:** Ming Jin.

**Methodology:** Ying-Xin Zi, Yu Deng, Mei-Qi Ji, Ya-Li Qin, Lu-Qi Nong, Zi-Qiang Liu, Ming Jin.

**Project administration:** Ming Jin.

**Resources:** Ying-Xin Zi, Yu Deng, Zi-Qiang Liu, Ming Jin.

**Software:** Ying-Xin Zi, Mei-Qi Ji, Zi-Qiang Liu, Ming Jin.

**Supervision:** Yu Deng, Ming Jin.

**Validation:** Ya-Li Qin, Lu-Qi Nong, Zi-Qiang Liu, Ming Jin.

**Visualization:** Ya-Li Qin, Lu-Qi Nong, Zi-Qiang Liu, Ming Jin.

**Writing – original draft:** Ying-Xin Zi, Yu Deng, Mei-Qi Ji, Ming Jin.

**Writing – review & editing:** Ying-Xin Zi, Yu Deng, Mei-Qi Ji, Ming Jin.

yingxin zi orcid: 0000-0002-0062-8725.
